# Biomedical applications of artificial exosomes for intranasal drug delivery

**DOI:** 10.3389/fbioe.2023.1271489

**Published:** 2023-09-07

**Authors:** Jinming Zhao, Jingxing Yang, Jian Jiao, Xiangdong Wang, Yan Zhao, Luo Zhang

**Affiliations:** ^1^ Department of Otolaryngology Head and Neck Surgery, Beijing Tongren Hospital, Capital Medical University, Beijing, China; ^2^ Beijing Laboratory of Allergic Diseases and Beijing Key Laboratory of Nasal Diseases, Beijing Institute of Otolaryngology, Beijing, China; ^3^ Department of Otorhinolaryngology, Beijing Youan Hospital, Capital Medical University, Beijing, China; ^4^ Department of Allergy, Beijing Tongren Hospital, Capital Medical University, Beijing, China; ^5^ Research Unit of Diagnosis and Treatment of Chronic Nasal Diseases, Chinese Academy of Medical Sciences, Beijing, China

**Keywords:** artificial exosomes, drug delivery, intranasal administration, blood-brain barrier, biomedical applications

## Abstract

Intranasal administration offers a feasible, non-invasive method of delivering therapeutic drugs to the brain, allowing therapeutic pharmaceuticals to be administered directly to the central nervous system by bypassing the blood-brain barrier. Furthermore, exosomes are naturally occurring cell-derived nanovesicles that can serve as carriers for a variety of chemical compounds. Many studies have focused on artificial exosomes as innovative medication delivery methods. As a result, trans-nasal delivery of artificial exosomes might be employed to treat brain illnesses in a novel method. This review will outline the drug delivery mechanism of artificial extracellular vesicles, emphasize its advantages as a nasal drug carrier, particularly its application as a novel nanocarriers in brain diseases, and focus on its prospective application in chronic inflammatory nose disorders. Finally, artificial exosomes may become a unique drug delivery mode for clinical therapeutic usage.

## Introduction

The blood-brain barrier (BBB) may be seen at the level of the cerebral microvasculature and is critical for maintaining homeostasis of the central nervous system (CNS) (Lochhead et al., 2020). The BBB significantly restricts access to all but tiny, nonpolar molecules, making medication penetration into the CNS challenging and limiting CNS illness therapy (Lochhead and Thorne, 2012). Intranasal drug administration is a feasible, noninvasive method of delivering therapeutic medications to the brain, and it has the potential to circumvent the BBB, allowing therapeutic compounds direct access to the CNS. Because it is noninvasive and may be repeated, intranasal injection of extracellular vesicles (EVs) has received a lot of attention (Kodali et al., 2019). While intranasal drug administration is an excellent method of delivering therapeutic medications, it has been demonstrated that there were some hurdles in transportation. Due to the permeability barrier in the nasal mucosa, the presence of mucociliary clearance system and the degradation of enzymes in the mucosa, multiple and complex factors contribute to many drugs into the brain is quite little by intranasal administration (Touitou and Illum, 2013; Kumar et al., 2017).

**FIGURE 1:**
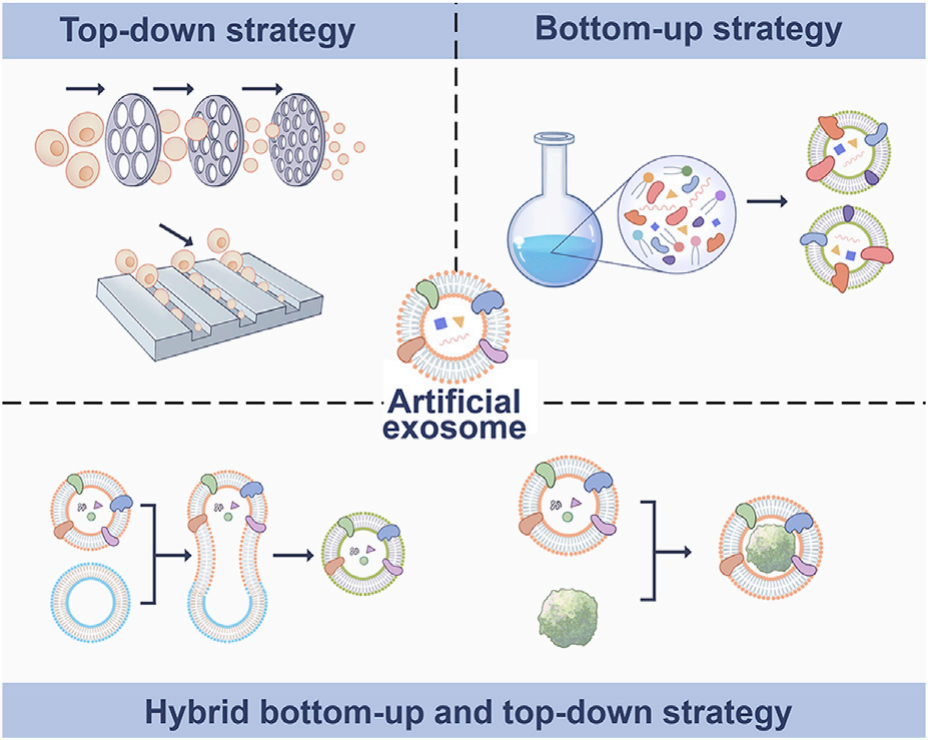
Synthetic methods of artificial exosomes. Artificial exosomes were obtained by top-down, bottom-up and biohybrid bottom-up and top-down strategies.

EVs are naturally occurring cell-derived nanovesicles that convey information between tissue microenvironments. They also have the ability to alter target cell activity and differentiation (Quesenberry and Aliotta, 2010; Hood and Wickline, 2012). EVs are found in most biological fluids, including blood, urine, saliva, cerebrospinal fluid, and breast milk (Zhang et al., 2019a). EVs can carry specific proteins, nucleic acid and metabolites, and the cargo in EVs retains its biological activity and can modulate recipient cells (Vázquez-Ríos et al., 2019). Although EVs are naturally derived vesicles, their surfaces can be conveniently modified, and surface engineering can confer targeted specificity to vesicles (Liang et al., 2021). The use artificial EVs is a new method for drug delivery. The nanovesicles (NVs), exosome mimics (EMs) and hybrid exosomes (HEs) are the major types of artificial EVs, which are obtained by top-down, bottom-up and biohybrid strategies, respectively (Figure 1). At present, top-down is the most widely used strategy to prepare artificial exosomes. The main advantage is that it contains being carried are from the producer cells, which mimics the biological complexity of natural exosomes. However, this method of production consumes more time and manpower, and the limited number of producer cells also limits production. The bottom-up strategy can produce artificial exosomes with pure composition and controllable characteristics, which can be used for large-scale production, significantly reducing time and labor costs. However, the biological complexity of synthetic exosomes is not as good as that of natural exosomes. The strategy of biohybrid has the advantage of including some natural components of exosomes, which may have higher delivery efficiency than liposomes and higher stability than exosomes. The limitation is that the yield will not be very high (García-Manrique et al., 2018; Li et al., 2021).

In recent years, a variety of artificial exosomes that can overcome the shortcomings of natural exosomes have been developed. It has been reported to be effective in the treatment of cancer (Zhang et al., 2019b; Lin et al., 2021), ischemic diseases (Aday et al., 2021) and injury (Staufer et al., 2021), and has a good prospect for clinical application. However, the research of this new biological treatment material is still in its early stage. In this review, we introduced the mechanism of artificial EVs delivery, reviewed its benefits as a drug carrier in nasal delivery, particularly its use as a new nanocarrier in brain diseases and emphasized the important potential for application in chronic rhinosinusitis and allergic rhinitis.

### In stroke/ischemia-reperfusion damage

**FIGURE 2:**
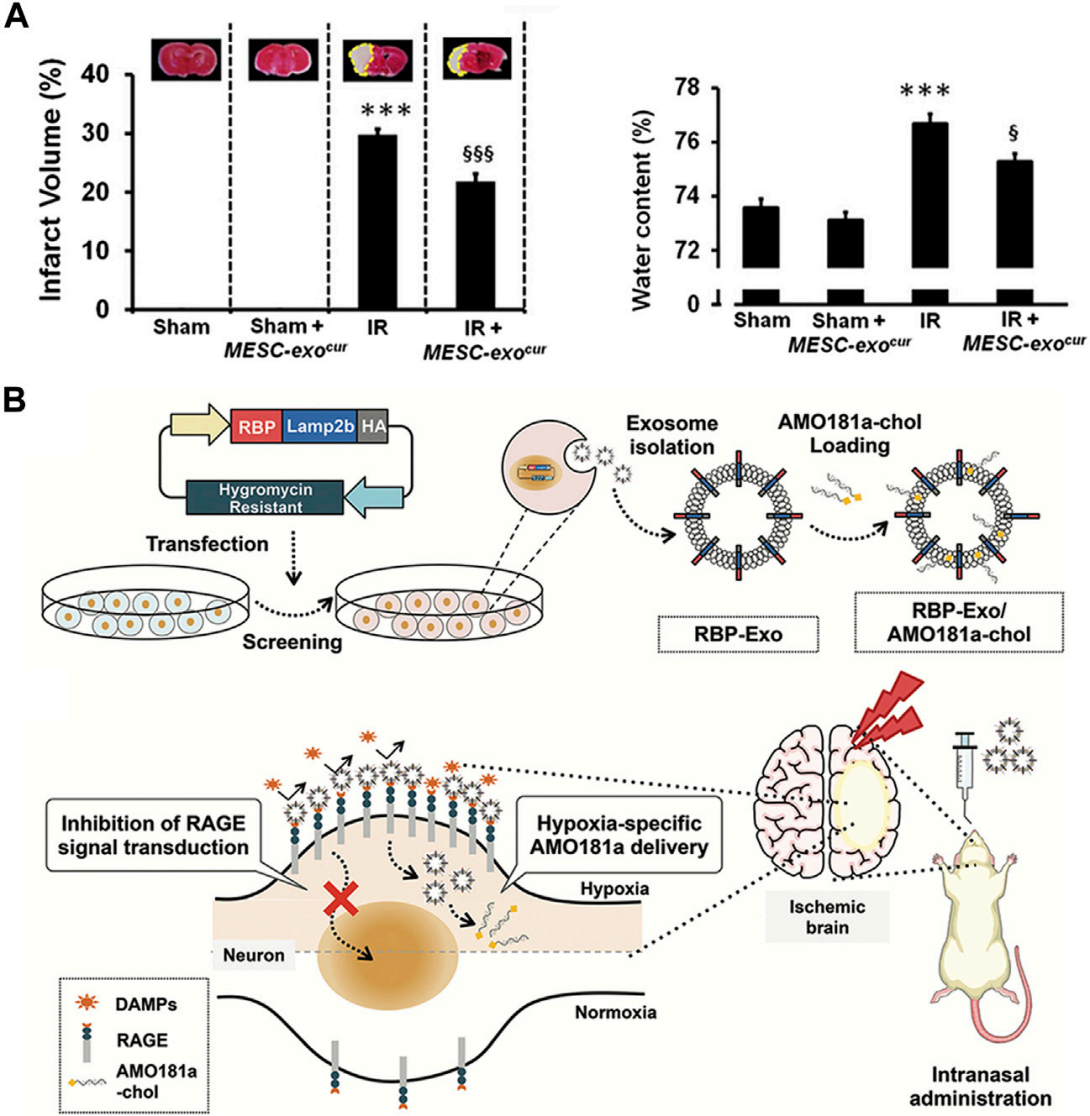
Biomedical applications of artificial exosomes for drug delivery in stroke/ischemia-reperfusion damage: **(A)** MESC-exo^cur^ treatment reduced infarct volume and water content in IR mice. Reproduced with permission from Kalani et al. (2016). **(B)** Production and anoxic-specific delivery of RBP-Exo/AMO181a-chol by intranasal administration. Reproduced with permission from Kim et al. (2021).

Stroke has a high fatality rate and a leading cause of disability (Towfighi and Saver, 2011). Ischemic stroke, a subtype of stroke, is caused by the narrowing of one or more cerebral arteries due to occlusion by emboli or thrombi. The disease is typically treated with thrombolytic drugs. However, brain injury often worsens after reperfusion. This pathological process is often called ischemia reperfusion (IR) injury (Zhou et al., 2018). In recent years, EVs produced from embryonic stem cells (ESCs) have been found to boost endogenous repair mechanisms and improve heart function after myocardial infarction and have shown therapeutic potential following stroke (Khan et al., 2015). Curcumin, as a natural polyphenol found in the rhizome of Curcuma longa (turmeric), has been extensively explored for its therapeutic effect ischemic stroke and its bioavailability is hampered by its low absorption and rapid metabolism (Li et al., 2017). One previous study created a curcumin-loaded EVs from cultured mouse embryonic stem cell (MESC) lines by a fast freeze-thawing technique and delivered nasally (Kalani et al., 2016). They found that MESC-exos^cur^ (curcumin-loaded in MESC derived exosomes) has greater stability and solubility and MESC-exos^cur^ therapy dramatically reduced neurological scores, cerebral edema, and infarct volume (Figure 2A).

Furthermore, receptor for advanced glycation end-products (RAGE) has been identified as a critical element involved in the pathophysiological process of IR injury (Ramasamy and Schmidt, 2012). Recombinant RAGE-antagonist peptide (RAGE-binding peptide, RBP) could have neuroprotective effect by reducing RAGE-mediated ischemic brain inflammation (Kim et al., 2021). Another study reported that delivery of anti-microRNA oligonucleotide (AMO) against miR181a (AMO181a) attenuated ischemic neuronal damage and that knockdown of miRN181a can reduce infarct volume. Therefore, investigators engineered an artificial EVs contains the RBP-linked exosomes (RBP-exos) combined cholesterol-modified AMO181a (AMO181a-chol) by hydrophobic interactions. The nanovesicles were administered intranasally to treat a mouse model of ischemic brain injury. As a result, intranasal administration of this hypoxia-specific vector allowed AMO181a to be delivered into the ischemic brain more effectively, and the RBP moiety promoted the delivery of AMO181a in the ischemic tissue. Amo181a-chol significantly inhibited RAGE pathways, inflammatory cytokines, apoptotic cells and infarct volume (Kim et al., 2021) (Figure 2B).

### In glioblastoma multiforme

**FIGURE 3:**
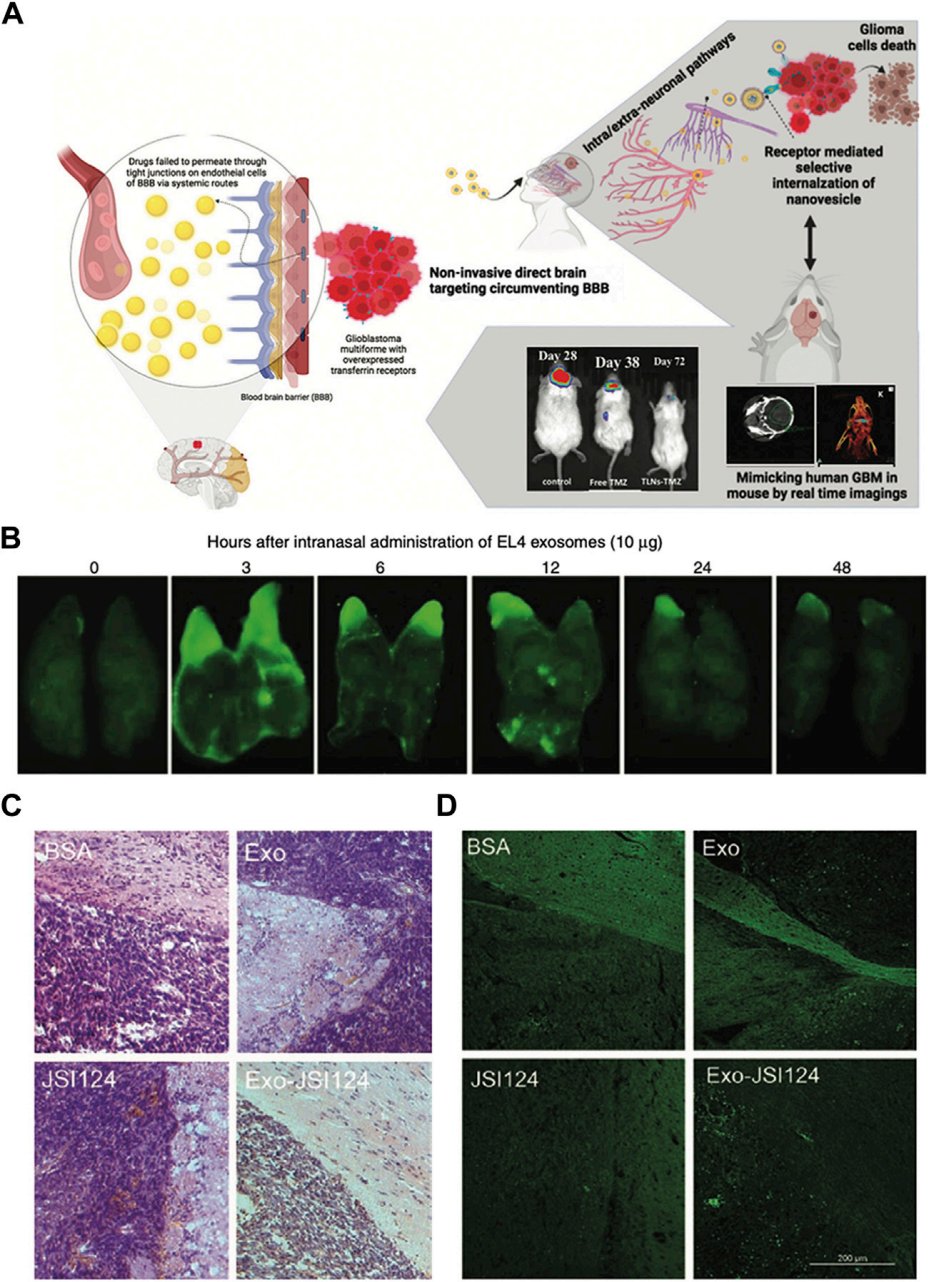
Biomedical applications of artificial exosomes for drug delivery in glioblastoma multiforme: **(A)** Schematic illustration of the synergistic drug-loaded transferrin-targeted nanovesicle system. The nanovesicles can bypass the blood-brain barrier and reach the brain through neurological pathways. Reproduced with permission from Sandbhor et al. (2021). **(B)** A total of 10 μg of IRDye 800-labeled EL-4 T cell-derived exosomes were administered intranasally into C57BL/6j mice, and the distribution pattern of exosomes in the brain was observed. The fluorescence of the brain was stronger after intranasal administration for 3 h. Reproduced with permission from Zhuang et al. (2011). **(C)** The results of HE-stained brain tumor tissue suggest that tumors adjacent to the olfactory region aggressively invaded the adjacent tissue of mice treated with BSA, Exo, and JSI124. In contrast, intranasally administration of Exo-JSI124 was much less invasive. Reproduced with permission from Zhuang et al. (2011). **(D)** The results of TUNEL staining were similar to those of HE staining. Reproduced with permission from Zhuang et al. (2011).

Glioblastoma multiforme (GBM) is the most malignant primary tumor of the central nervous system, having a poor prognosis and a high mortality rate. Chemotherapy remains the primary therapeutic choice for GBM treatment and the existence of the BBB inhibits medication effects. Sandbhor et al. (2021) recently shown in orthotopic GBM mouse models that *in situ* administration of hydrogel embedded with miltefosine (HePc, a proapoptotic antitumor agent) and temozolomide (TMZ, a DNA methylating agent)-loaded targeted nanovesicles could suppress the tumor relapses. Because transferrin receptors are overexpressed on the surface of the tumor, surface engineering of vesicles can increase cellular uptake and medication internalization into tumor (Zhao et al., 2020). As a result, TMZ was given via lipid nanovesicles (LNs) that were surface transferrin-decorated and coencapsulated with HePc. In terms of tumor-bearing mouse survival, the effectiveness of the TLNs rose by 1.8-fold when compared to free medicines. Furthermore, off-target organ damage has been demonstrated in the clinic, and tailored nanovesicles in conjunction with HePc will be tolerated better than traditional systemic delivery, lowering systemic drug exposure. This study shows that LNs have the potential to boost brain medication bioavailability following intranasal delivery, and the treatment strategy is also likely to improve compliance in patients on long-term drug therapy (Sandbhor et al., 2021) (Figure 3A).

The signal transducer and activator of transcription 3 (STAT3) protein is constitutively active in several forms of human malignancies and is essential for tumor development, including GBM. Cucurbitacin I (JSI-124), as a potent STAT3 signaling pathway inhibitor, have antitumor properties in human breast cancer, neuroblastoma, lung cancer, murine melanoma cell lines and B cell leukemia (Ren et al., 2014). Furthermore, JSI-124 inhibits the proliferation of glioblastoma polymorphic cells by increasing apoptosis and inducing G_2_/M-phase cell cycle arrest (Su et al., 2008). Therefore, to investigate the therapeutic impact of artificial EVs, GL26 brain tumor model mice were treated with the exosome-encapsulated JSI-124 (Exo-JSI124). On the one hand, EVs have been observed in brain tissue to selectively target microglial cells, exhibiting potent anti-tumor capabilities (Figures 3B–D). On the other hand, Exo-JSI124-treated mice had considerably higher survival periods, with an average of 44.5 days. Furthermore, none of the mice displayed any signs of toxicity or aberrant behavior during the 15-day period after Exo-JSI124 intranasal administration (Zhuang et al., 2011).

In a recent work, researchers used a pressure-based disruption and reconstitution procedure to load miRNA (anti-miRNA 21 and miRNA 100) while managing the size of microfluidically processed EVs (mpEVs) (Wang et al., 2021). In this work, vesicles were collected from neural stem cells, designed to overexpress the CXCR4 receptor, and then engineered to have the ideal size of mpEVs. Intranasal injection of miRNA-loaded mpEVs to a GBM mouse model resulted in a consistent pattern of mpEV transport across the nasal epithelium, bypassing the BBB and entering the cerebral compartment. Such EVs have a GBM-specific tropism, and the loaded miRNA made GBM cells susceptible to TMZ. The findings revealed that the miRNA-loaded mpEVs not only reduced tumor size but also increased mouse survival.

### In brain inflammatory illness and other brain diseases

**FIGURE 4:**
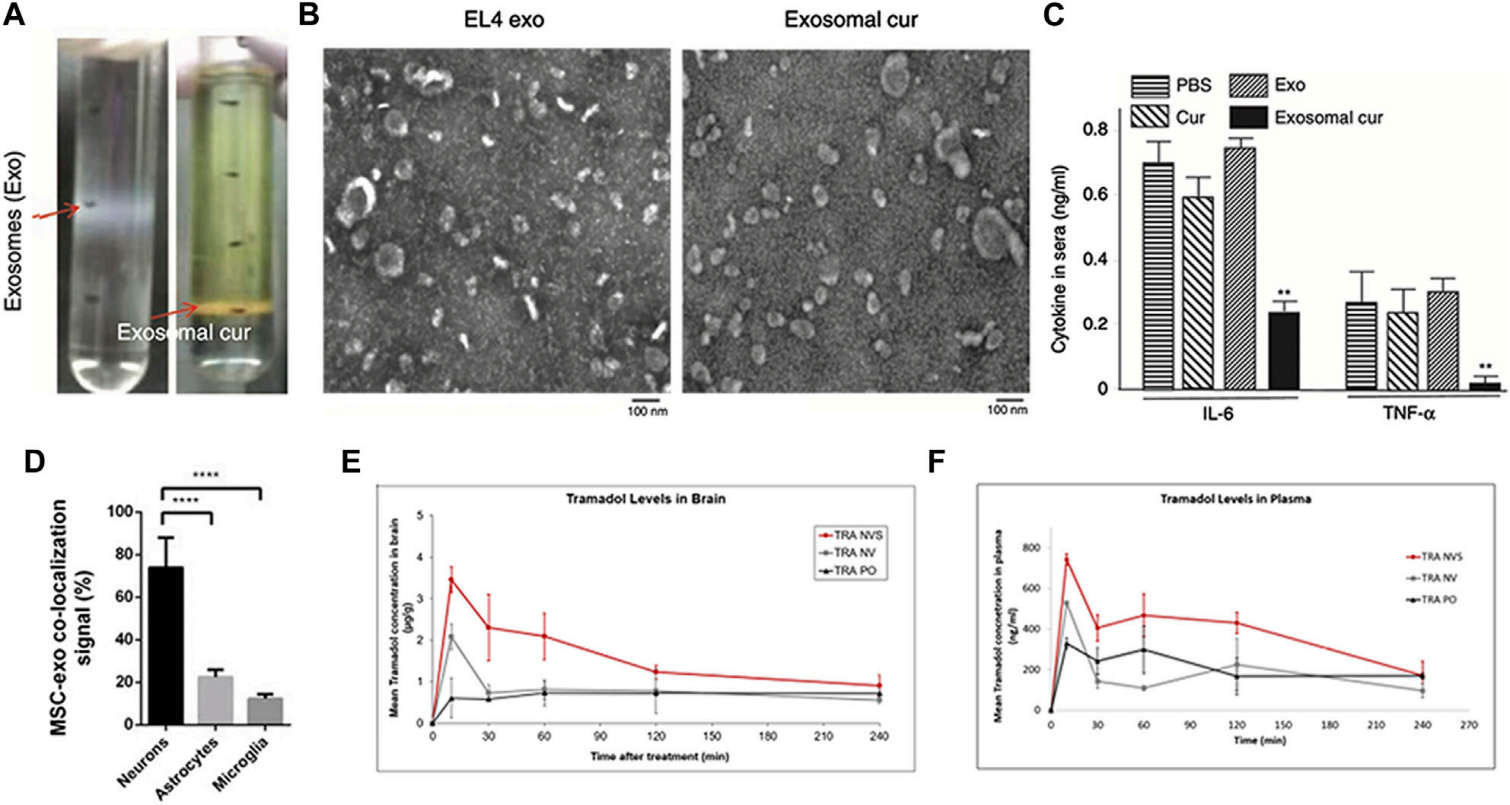
Biomedical applications of artificial exosomes for drug delivery in brain inflammatory illness and other brain diseases: **(A)** Exo-cur was obtained by gradient centrifugation after co-incubation of curcumin and EL-4 exosomes. **(B)** Electron microscopic images of EL-4 exosomes and Exo-cur. **(C)** Exo-cur can reduce IL-6 and TNF-α secretion after LPS stimulation. Reproduced with permission from Sun et al. (2010). **(D)** Nasal administration of MSC-Exo in SCI rats showed that MSC-Exo inherit MSC targeting capability, rendering them a suitable delivery system to the injured spinal cord. Reproduced with permission from Guo et al. (2019). **(E)** Pharmacokinetic profiles of tramadol in the brain and plasma **(F)** following administration of the drug in the nasal nanovesicular system (TRA NVS) compared to the nasal nonvesicular system (TRA NV) and oral administration (TRA PO). The TRA PO and TRA NV groups showed lower levels than the NVS-treated animals. Reproduced with permission from Touitou et al. (2021).

It is evident that microglial cells, or brain-resident macrophages, play an important role in many CNS inflammation-related disorders, including meningitis, migraine headaches, Parkinson’s disease, and others. Curcumin, as previously said, not only has anticancer and preventative effects, but it is also a potent anti-inflammatory agent. After incubation, the mixture was centrifuged using a sucrose gradient to extract exosomal curcumin (Exo-cur), the size and morphology of Exo-cur were similar to those of natural exosomes (Figures 4A, B). LPS-challenged mice were treated with intranasal treatment of exosome-encapsulated curcumin. It was discovered in the brain 1 h after the fake EVs were administered, which is consistent with prior findings. The levels of cytokines decreased after treatment (Sun et al., 2010) (Figure 4C). Meningitis is a disease caused by microbial infection that can be divided into bacterial meningitis, viral meningitis, tuberculosis meningitis and cryptococcal meningitis. Bacterial meningitis responsible for 75% of all meningitis cases globally and the strong dosages of antibacterial medications are used to treat the infection (van de Beek, 2012). The key to efficient meningitis management is to keep high doses of antibiotics in the brain. Ofloxacin-loaded transfersomal nanovesicles (OFLOX-TNVs) were created for the treatment of meningitis by nasal administration to avoid the difficulties of its high-dose use. These NVs were created using the thin film hydration process with chemicals such as lecithin, edge activator (EA), and cholestasis. These findings imply that OFLOX-loaded nanometastases might be a useful colloidal delivery mechanism for brain targeting and treatment of bacterial meningitis (Hussein et al., 2019).

Spinal cord injury (SCI) is a crippling disorder with few treatment options and erratic recovery. The inherent obstacle to axonal development in the pathological phase of SCI is mostly phosphatase and tensin homolog (PTEN). PTEN downregulates cytoplasmic mammalian target of rapamycin (mTOR) activity and plays an important role in regulating the regeneration of corticospinal neurons (Terenzio et al., 2018). MSC-Exos may be able to cross the BBB and move to the damaged spinal cord region (Figure 4D). In another animal studies, this type of EV has been shown to greatly increase mobility, sensory recovery, and urine reflex recovery (Guo et al., 2019).

A nasal nanovesicle delivery system (NVS) for pain treatment was created and developed in a recent study. Ketoprofen (KET), butorphanol (BUT), or tramadol (TRA) were incorporated in a phospholipid nanovesicular carrier in an animal model of pain, resulting in a faster onset and improved analgesic effectiveness (Touitou et al., 2021). According to pharmacokinetic results, the blood concentration of the ketoprofen nasal nanovesicular system (KET-NVS) after administration was higher than that of the drug after oral administration, the delivery time was shorter, and it took less time to reach the highest plasma concentrations (Figures 4E, F). This phenomenon is also consistent in the tramadol nasal nanovesicular system (TRA-NVS). Taken together, the NVS not only offers a high transmission efficiency but is also safe for the nasal mucosa. In conclusion, this noninvasive, rapid, cell-free, lesion specific, and effective therapy has a lot of potential for therapeutic usage in brain disorders.

### In chronic rhinosinusitis and allergic rhinitis

**FIGURE 5:**
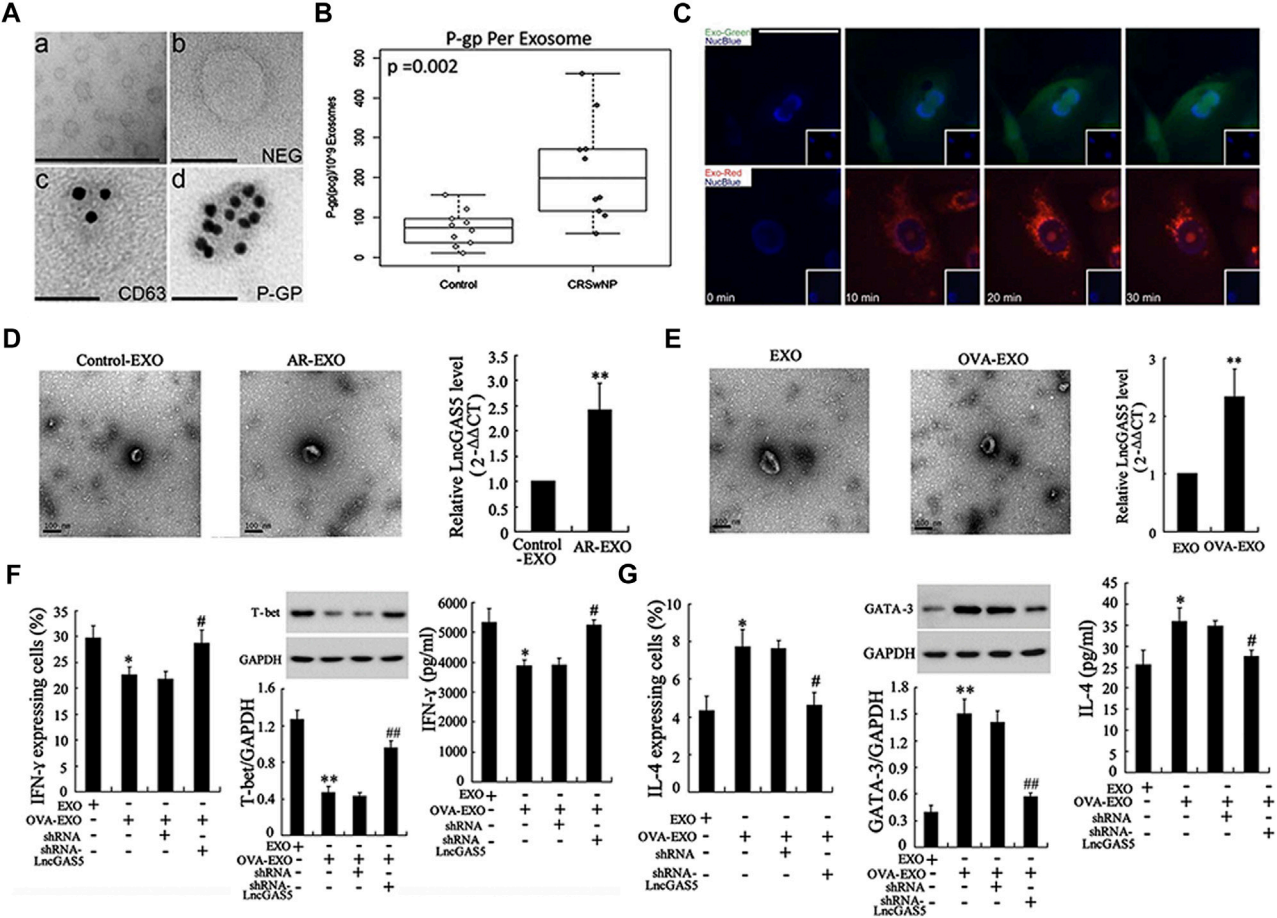
Research progress of exosomes in chronic rhinosinusitis and allergic rhinitis: **(A)** transmission electron microscopy images showed exosomes purified from nasal mucus **(i)** and negative controls **(ii)**, immunolabeling of exosome marker CD63 **(iii)**, and P-gp **(iv)** localization on exosome membranes. **(B)** The concentration of P-gp per exosome was significantly higher in patients with CRSwNP compared to the control group. **(C)** Time-lapse fluorescent images showed rapid absorption of labeled exosomal proteins and RNA within 10 min after exposure to purified exosomes. Reproduced with permission from Nocera et al. (2017). **(D)** LncGAS5 was upregulated in both exosomes of AR mucosa (AR-EXO) and OVA-treated RPMI 2650 cells (OVA-EXO) **(E)**. **(F)** LncGAS5 in OVA-EXO suppresses Th1 differentiation via regulating EZH2 and T-bet. **(G)** LncGAS5 in OVA-EXO promotes Th2 differentiation via regulating EZH2 and T-bet. Reproduced with permission from Zhu et al. (2020).

Chronic rhinosinusitis (CRS) is a common clinical inflammatory disease of the nasal cavity and sinuses that often involves multiple sinuses (Stevens et al., 2015). The pathogenesis of CRS is complicated, and many internal and external factors influence its occurrence and development. EVs have also been found to play an important role in chronic sinusitis. Nasal mucus-derived EVs (NMDEs) containing cystatin-SA (CST-2) can predict the severity and phenotype of the CRS phenotype (Miyake et al., 2019). Pappalysin-1 (PAPP-A), an EVs biomarker, can not only monitor the severity of CRS but also predict recurrence at an early stage (Mueller, 2021). Previous study revealed that the nasal microbiota in patients with CRS had increased abundance and reduced diversity (Choi et al., 2014), and that bacterial-derived EVs may be responsible for the onset of CRS and inflammation (Miyake et al., 2019). LPS exposure *in vivo* and *in vitro* induced a 2-fold increase in NMDEs secretion (Nocera et al., 2019). P-glycoprotein (P-gp) was enriched in CRSwNP NMDEs and those NMDEs are capable of rapid interepithelial protein transfer (Nocera et al., 2017) (Figures 5A–C). These EVs not only exhibit direct microbiocidal activity but also actively arm epithelial cells with immunoprotective proteins that can be used to defend against the same microorganisms in mucus.

Allergic rhinitis (AR) is one of the most common upper respiratory disorders worldwide, with a convoluted cause. It is a nasal mucosal inflammatory condition induced by allergen exposure. The imbalance between Th1 and Th2 differentiation is implicated in the development of AR (Eifan and Durham, 2016). Recent research suggests that EVs may have a role in immune-driven illness such as allergies. MiRNA profiles in AR patients’ EVs were significantly changed as compared to healthy controls. Furthermore, considerable enrichment of these differentially expressed miRNAs in certain biological and physiological processes, such as the B cell receptor signaling, natural killer cell-mediated cytotoxicity, and T cell receptor signaling pathway, have been established (Hovhannisyan et al., 2021). Another study found that miR-146a-loaded nasal epithelial cell (NEC)-derived EVs increased IL-10 production in monocytes, which inhibited downstream allergy reactions. IL-10+ monocytes exert immunosuppressive effects on CD4^+^ effector T cells and Th2 polarization in this mouse AR model (Zhou et al., 2021). GAS5, a long noncoding RNA (lncRNA), is expressed in nasal mucus-derived EVs as well as ovalbumin-stimulated NEC-derived EVs in AR (Figures 5D, E). And EVs loaded with the lncRNA GAS5 could inhibit Th1 cell development while inducing Th2 differentiation (Zhu et al., 2020) (Figures 5F, G).

## Conclusion and perspectives

EVs have the potential to overcome biological barriers by themselves, and their ability to express particular proteins, cell adhesion molecules, or ligands that cause natural target selectivity for certain recipient cells is what makes them appealing as delivery vehicles. Artificial EVs for intranasal administration have been widely used as vectors for delivering therapeutic drugs in some kinds of brain illness. However, its transmission efficiency still needs to be further improved. The intranasally administered drug enters the parenchymal space of the brain or cerebrospinal fluid (CSF) via trigeminal and olfactory nerves via axonal or endocytic routes and intra- or extra-neuronal pathways, respectively (Djupesland PG and Mahmoud, 2014). Although nasal administration bypasses the BBB, the mucosal barrier still important in delivery. The medicine must be deposited on the luminal surface of the epithelial membrane and absorbed before being removed or destroyed in the respiratory system. Controlling the drug’s release profile as it passes through different biological barriers may also be required for adequate absorption (Ghadiri et al., 2019).

Many artificial approaches, such as *in situ* chitosan hydrogels, mucoadhesive nanostructured lipid carriers, and chitosan nanoparticles, have been used to overcome the nasal mucosa barrier and improve the efficacy of drug therapy. However, several of these techniques may cause nasal toxicity and membrane component leaching, resulting in local irritation of the nasal mucosa. Full artificial EVs based on phospholipids have been developed for nasal delivery. This carrier has demonstrated the potential to improve drug delivery to the brain and the systemic circulation without generating nasal mucosal irritation or toxicity. Furthermore, the carrier combining magnesium ions with soft phospholipid vesicles and a mucoadhesive molecule called the phospholipid magnetosome was performed to explore a transport carrier with higher efficiency (Natsheh and Touitou, 2018). This chemically modified nasal carrier can boost pharmacological effects, probably by a combination of absorption enhancement and prolongation of mucosal contact. It seems to solve the difficulties caused by the BBB and mucosal barrier, but its safety and other aspects are still being studied as a new method.

Although several studies demonstrated that nasal administration of artificial nanovesicles had significant advantages in safety and transport efficiency on the treatment of brain illness. Collectively, the BBB is considered to be the main barrier to drug penetration into the CNS. Intranasal administration is an attractive alternative route to central nervous system administration because it bypasses the BBB more effectively than systemic administration. Artificial EVs can effectively cross the BBB. Intranasal administration of artificial EVs may be particularly appropriate for drugs that cannot achieve perfect blood concentrations in the brain due to the presence of the BBB, drugs that tend to cause adverse effects in peripheral tissues or in the blood, and drugs that are easily degraded by intravenous or oral administration. Although intranasal treatment has been shown to be effective in many brain illnesses, it has not been used in chronic inflammatory nose disorders. Only few reports on the use of artificial EVs in the treatment of chronic nasal diseases, such as chronic rhinosinusitis and allergic rhinitis. The great ability of artificial EVs in nasal disorders of the nasal cavity can be further explored in the future.
